# Fatty acids and recurrence of major depressive disorder: combined analysis of two Dutch clinical cohorts

**DOI:** 10.1111/acps.13136

**Published:** 2019-12-26

**Authors:** C. S. Thesing, A. Lok, Y. Milaneschi, J. Assies, C. L. H. Bockting, C. A. Figueroa, E. J. Giltay, B. W. J. H. Penninx, H. G. Ruhé, A. H. Schene, M. Bot, R. J. T. Mocking

**Affiliations:** ^1^ Department of Psychiatry Amsterdam Public Health Research Institute Amsterdam UMC Vrije Universiteit Amsterdam Amsterdam The Netherlands; ^2^ Department of Psychiatry Amsterdam Public Health Research Institute Amsterdam UMC Academisch Medisch Centrum University of Amsterdam Amsterdam The Netherlands; ^3^ Department of Psychiatry Leiden University Medical Center Leiden The Netherlands; ^4^ Department of Psychiatry Radboud University Medical Center Nijmegen The Netherlands; ^5^ Donders Institute for Brain, Cognition and Behavior Radboud University Medical Center Nijmegen The Netherlands

**Keywords:** major depressive disorder, recurrence, omega‐3, omega‐6, docosahexaenoic acid

## Abstract

**Objective:**

Omega‐3 (n‐3) and omega‐6 (n‐6) polyunsaturated fatty acid (PUFA) alterations in patients with major depressive disorder (MDD) have been shown to persist after remission. Whether these alterations are risk factors for MDD recurrence remains unknown. Here, we examined whether fatty acids predict time until MDD recurrence in remitted MDD patients.

**Methods:**

Data were used from remitted MDD patients of the Netherlands Study of Depression and Anxiety (*n* = 356) and the Depression Evaluation Longitudinal Therapy Assessment studies (*n* = 118). Associations of FAs with time until MDD recurrence up to 8‐year follow‐up were analyzed using Cox regression analyses. Study‐specific estimates were pooled using mega‐ and meta‐analysis techniques.

**Results:**

27.5% (NESDA) and 56.8% (DELTA) participants had an MDD recurrence. Pooled results showed that no FA was significantly associated with time until MDD recurrence (n‐3 PUFAs: hazard ratio (HR) = 1.17, 95% confidence interval (CI) = 0.98–1.41, *P* = 0.082; n‐6 PUFAs: HR = 1.08, 95% CI = 0.84–1.38, *P* = 0.55).

**Conclusion:**

In remitted MDD patients, circulating PUFAs were not associated with prospective risk of MDD recurrence. Consequently, circulating PUFAs are unlikely to reflect a vulnerability marker for recurrence, so correcting n‐3 PUFA ‘deficits’ through supplementation does not seem a promising option to prevent MDD recurrence.

**Significant outcomes**

- Although PUFA alterations in depressed patients have been shown to persist after remission, these were not associated with prospective risk of MDD recurrence.
- As circulating PUFAs are unlikely to reflect a vulnerability marker for disease recurrence, correcting n‐3 PUFA ‘deficits’ through supplementation does not seem a promising option to prevent MDD recurrence.
- Higher EPA and, although not statistically significant, other PUFAs were associated with a higher risk of recurrence, suggesting that alterations during remission may alternatively reflect an adaptive process.

**Limitations**

- Although MDD recurrence was assessed repeatedly during follow‐up, recurrences were assessed retrospectively, and may therefore be influenced by memory bias.
- Fatty acids in NESDA and DELTA were measured using two different assays in the two different study samples; however, by running meta‐analyses with random effects, we acknowledged clustering of measures within cohorts.
- EPA levels were missing in the NESDA study, and data on use of n‐3 PUFA supplements were missing in the first DELTA cohort study.

## Introduction

Major depressive disorder (MDD) is an important mental health issue with a high prevalence and recurrence rate 1. A mechanism of interest in the (etio)pathophysiology of MDD involves fatty acid (FA) metabolism, including the nutrients omega‐3 (n‐3) [e.g., eicosapentaenoic acid (EPA) and docosahexaenoic acid (DHA)] and omega‐6 (n‐6) polyunsaturated fatty acids (PUFAs) [e.g., arachidonic acid (AA)]. N‐3 and n‐6 PUFAs are called essential FAs as humans lack synthesizing capacity and must ingest them through their diet or supplements 2. The most important dietary source of n‐3 PUFAs is fatty fish, but >90% of people do not meet dietary intake recommendations 3, 4, 5.

Fatty acids are thought to be involved in the psychopathology of MDD in various ways: via dysregulation of biological stress systems 6, 7, including the immune‐inflammatory system 8 and the hypothalamic–pituitary–adrenal axis 6, 9; and via cell membrane fluidity, functioning of membrane‐bound receptors 10, oxidative stress 7, 10, 11, and mood related neurotransmitter metabolism 12. Of note, n‐3 and n‐6 PUFAs often exert opposite effects; for example, n‐3 PUFAs are generally considered anti‐inflammatory, while n‐6 PUFAs have pro‐inflammatory effects.

Cross‐sectional epidemiological and case–control studies suggest that current MDD is associated with low n‐3 PUFA intake through food (including low fatty fish intake) 13, 14, 15 and low n‐3 PUFA concentrations in plasma, erythrocyte membrane, and postmortem brain tissue 14, 16, 17, 18, 19, 20, when compared to non‐depressed controls. In addition, supplementation of n‐3 PUFAs may exert antidepressant effects in MDD 18, 21, 22, 23, although the heterogeneity between studies is large with several trials showing no significant effect. Furthermore, alterations in overall FA metabolism have been associated with MDD, expressed by indices that delineate main structural FA characteristics (i.e., the unsaturation index and the carbon chain length index) 9, 19. After remission of MDD, FA alterations have been noted by some 24, 25 but not all studies 20.

Furthermore, n‐3 PUFA levels and intake have been prospectively linked to onset of a new MDD episode in a mixed sample of participants with and without a history of MDD. Three meta‐analyses of longitudinal observational studies showed that a high quality of the diet and a high fish intake (which is high in n‐3 PUFAs) were associated with a lower incidence of new‐onset depression 26, 27, 28. However, Molendijk et al. (2018) found that studies that adjusted for depression severity at baseline or that used a formal MDD diagnosis as outcome did not yield statistically significant findings. Moreover, higher baseline n‐6:n‐3 PUFA ratios in the blood predicted prospective onset of MDD over 7 years in young people with at‐risk mental states 29. However, conflicting evidence exists. Thesing et al. (submitted) and others found no association between FA levels and prospective depression risk in samples including both participants with and without a history of MDD 30, 31. These inconsistencies may be explained by differences in study samples (e.g., age and sex differences, with vs. without MDD history), FA assessments (e.g., individual FA vs. FA profiles), and outcomes (e.g., first episode onset vs. recurrence, dichotomous MDD recurrence [yes/no] vs. time until MDD recurrence).

Despite these inconsistent findings, intervention studies have tested whether n‐3 PUFA supplementation is effective in the prevention of MDD onset 32, 33. For example, the MooDFOOD study found no preventive effect of multi‐nutrient supplementation, including n‐3 PUFAs, on the onset of MDD during 1 year in over 1000 subclinically depressed and overweight persons with and without a history of MDD 32. While focusing specifically on remitted MDD patients, Antypa et al. (2012) did find that n‐3 PUFA supplementation improves emotional cognition and mood 33.

In conclusion, n‐3 PUFA supplementation to correct FA alterations observed during an MDD episode may have antidepressant effects, but data on the prospective association between FAs and the onset of new MDD episodes remain contradictory. Whether high n‐3 PUFA levels can prevent MDD onset or recurrence therefore remains questionable. This contradictory prospective evidence contrasts with the observation that (I) fish oil is the (non‐vitamin/non‐mineral) dietary supplement most commonly taken by both adults and children 34, also with the idea to prevent depression; and with (II) the initiation of new preventive intervention studies trying to delay depression recurrence using n‐3 PUFAs. Interestingly, evidence suggests that FA alterations in MDD persist during remission, suggesting (endophenotypic) traits, and it has been hypothesized that these alterations are involved in vulnerability for subsequent MDD episodes. However, designs of previous studies were not optimal due to the use of antidepressant prescriptions being indicative for relapse, instead of structured interviews for MDD relapse 30, the use of specific samples (i.e., postmenopausal women 31), or the use of dichotomous outcome measures for MDD recurrence (yes/no) instead of more detailed measures such as the time until MDD recurrence 31.

### Aims of the Study

The aim of our study was to provide more decisive evidence on the prospective relation between omega‐3 and omega‐6 polyunsaturated fatty acid measures and time until recurrence of major depressive disorder in remitted major depressive disorder patients. Focusing on remitted major depressive disorder patients only is important as mechanisms of disease recurrence in remitted patients may be different from mechanisms of disease onset in participants without a history of major depressive disorder. Using a large sample of two detailed, unique, longitudinal datasets, we addressed the hypotheses that low omega‐3 levels, low eicosapentaenoic levels, low docosahexaenoic levels, low omega‐3‐to‐omega‐6 ratios, shorter fatty acid carbon chain length and a lower degree of unsaturation, and high omega‐6 levels predict a faster recurrence of major depressive disorder in remitted major depressive disorder patients.

## Materials and methods

For the present analyses, data of two studies were used: the Netherlands Study of Depression and Anxiety (NESDA) and the first and second Depression Evaluation Longitudinal Therapy Assessment (DELTA) cohort studies. The study samples, measurements, and statistical analyses are described below for each study.

### Study samples and outlines

#### Study sample and outline of the NESDA study

Between 2004 and 2007, 2981 participants aged between 18 and 65 years were recruited from the Dutch general population (19%), primary health care (54%), and specialized mental health care (27%) to participate in the NESDA study, an ongoing longitudinal observational cohort study on the predictors, courses, and consequences of depression and anxiety 35. The research protocol was approved by the ethics committees of participating universities. All respondents provided written informed consent. The study was carried out in accordance with the latest version of the Declaration of Helsinki. Exclusion criteria were a poor comprehension of the Dutch language and having a primary clinical diagnosis of another (e.g., psychotic, obsessive–compulsive, bipolar, or severe addiction) disorder. For this study, baseline, 2‐, 4‐, and 6‐year follow‐up data were used. Participants provided blood samples at baseline (after instructions for overnight fast) and underwent a psychiatric interview at baseline and all follow‐up periods during which the presence of depression was assessed. Additional criteria for participants to be included in the analyses in the present study were as follows: (i) having a lifetime diagnosis of MDD but no current diagnosis of MDD at baseline (e.g., depression in remission for at least 1 month) according to the Composite International Diagnostic Interview (CIDI, version 2.1) 36 and (ii) a baseline Inventory of Depressive Symptomatology Self‐Report score of ≤13 (cutoff for mild symptoms). This resulted in 362 eligible remitted MDD patients at baseline. We excluded six participants (1.7%) due to missing blood samples, resulting in 356 NESDA participants (final sample size).

#### Study sample from the DELTA studies

The Depression Evaluation Longitudinal Therapy Assessment (DELTA) study started as a randomized controlled trial (DELTA‐RCT), which formed the basis of two extended cohort studies. The RCT compared the efficacy of preventive cognitive therapy on MDD relapse and recurrence 37. The extended cohort studies investigated underlying mechanisms of recurrence, among others with neuroimaging 25, 38. For all studies, written informed consent was obtained after complete description of the study to the participants. The protocols were approved by the relevant institutional ethics review committees.

In the first DELTA cohort study, between February 2002 and September 2002, 88 participants could be recruited at two years of follow‐up of the DELTA‐RCT. They had to be in remission for ≥10 weeks and have a 17‐item Hamilton Depression Rating Scale (HDRS) ≤10 39. In the second cohort study (2011–2017), an additional 51 remitted MDD patients were included in the DELTA‐neuroimaging study. Patients were recruited through advertisements in freely available online and house‐to‐house papers, posters in public spaces, and from previous studies in our and affiliated research centers.

Selection criteria to participate in the current analyses for both DELTA cohort studies were (i) ≥2 MDD episodes in the past, as defined according to the Diagnostic and Statistical Manual of Mental Disorders (DSM‐IV; American Psychiatric Association, 1994) and assessed with the Structured Clinical Interview for DSM‐IV (SCID) 40 administered by trained interviewers, and (ii) a 17‐item HDRS > 7 (a cutoff often used for mild symptoms) 39, 41. Exclusion criteria were (hypo)mania or a history of bipolar disorder, any lifetime or current psychotic disorder, organic brain damage, alcohol or drug misuse, predominant anxiety disorder, and recent electroconvulsive therapy 37. The DELTA‐neuroimaging study additionally excluded patients with severe personality disorder (SCID); antidepressant use; standard MRI exclusion criteria (e.g., metal objects in the body, claustrophobia); and severe general physical illness.

Of all DELTA participants (*n* = 139), 18 participants had no complete blood measurement and 3 participants had no data on time until recurrence of MDD, resulting in 118 eligible DELTA participants for analyses (final sample size). Follow‐up time for the first DELTA cohort study was 8 years and for DELTA‐neuroimaging 28 months. In analyses, both DELTA studies were considered as one study as measurements and in‐ and exclusion criteria were highly comparable.

### PUFA assessment

The following PUFAs were measured in all two studies at baseline: n‐3 PUFA, DHA, n‐6 PUFA, n‐3:n‐6 PUFA ratio, FA carbon chain length index, and degree of unsaturation index. EPA was measured only in the DELTA study. To give an impression on how all fatty acid measures are interrelated, Tables S1 and S2 show the Spearman correlations between all fatty acid measures in NESDA and DELTA respectively.

#### PUFA assessment in NESDA

A detailed description of PUFA assessment in the NESDA study can be found elsewhere 20. The fatty acids measured in NESDA are esterified fatty acids stemming from the lipoprotein particles, so bound within cholesteryl esters, triglycerides, and phospholipids inside lipoproteins particles. They were assessed in EDTA plasma samples which were stored at −80°C for later assessment. At baseline, blood samples were shipped in 2 batches (April and December 2014, further referred to as metabolic assessment waves 1 and 2 respectively). In 2015, PUFA levels were quantified at 22°C using a commercially available high‐throughput proton Nuclear Magnetic Resonance (NMR) metabolomics platform (Nightingale Health Ltd., Helsinki, Finland) 42. Fatty acid measures included in this study were n‐3 PUFA, DHA, n‐6 PUFA, n‐3/n‐6 PUFA ratio, FA carbon chain length index (CLI), and degree of unsaturation index (UI). Fatty acid concentrations were expressed in mmol/l. The CLI and the UI were calculated at the extensively validated metabolomics platform Nightingale Health Ltd. 42.

#### PUFA assessment in the DELTA studies

A detailed description of PUFA assessment in the DELTA studies can be found elsewhere 38, 43. FAs were measured in washed erythrocytes and were analyzed using capillary gas chromatography. Within 4 h of collection, plasma was separated and stored at −80°C until analysis. FA concentrations or indices included in this study were n‐3 PUFAs, DHA, EPA, n‐6 PUFAs, n‐3/n‐6 PUFA ratio, CLI, and UI. The levels of five measured n‐3 PUFAs and eight measured n‐6 PUFAs were added up to calculate the total n‐3 PUFA and n‐6 PUFA levels respectively. Levels were expressed in pmol/10e6 erythrocytes. Additionally, data were available on levels of one n‐5 PUFA, two n‐7 PUFAs, and five n‐9 PUFAs. The CLI, which provides information about FA carbon chain length, and the UI, which indicates the number of double bonds per FA, were calculated as described earlier 24.

### Time until recurrence of MDD

#### Time until recurrence of MDD in NESDA

Time to onset of a new depressive episode (up to 6‐year follow‐up) was determined with the CIDI in combination with the Life Chart Interview (LCI) 44. If a participant fulfilled the criteria of a depressive disorder diagnosis during any of the follow‐up periods (every 2 years), the LCI was used as a calendar method to determine life events during the 2‐year follow‐up period to refresh memory, and subsequently asked the months during which two core depressive symptoms (i.e., sad and depressive feelings or loss of interest) were present. LCI data were used to define the time to first depressive episode with at least minimal burden. Participants lost to follow‐up or without recurrence during follow‐up were considered censored at the last follow‐up time point with available data or at the end of the total follow‐up period respectively. A dichotomous variable was made indicating whether a recurrence had taken place (1) or not/whether the patient was censored (0).

#### Time until recurrence of MDD in the DELTA studies

In the first DELTA cohort study, time until recurrence of MDD (in months) from baseline until the end of the study (up to 8‐year follow‐up) was assessed using the SCID, which was administered at three follow‐up time points (at 1, 3.5, and 8 years). In the second DELTA cohort study (DELTA‐neuroimaging)*,* to prospectively assess recurrence, follow‐up assessments of remitted MDD participants were conducted every 4 months up until 28 months after baseline by phone 38. The occurrence of MDD and its start date were assessed by the SCID. Participants lost to follow‐up or without recurrence during follow‐up were considered censored at the last follow‐up time point with available data or at the end of the total follow‐up period respectively. A dichotomous variable was made, indicating whether a recurrence has taken place (1) or not/whether the patient was censored (0).

### Covariates and effect modifiers

In NESDA, analyses were adjusted for metabolic assessment wave (1 or 2) to account for any differences between the shipments of the blood samples. For the DELTA studies, analyses were adjusted for study (first or second cohort study) to account for any possible differences potentially induced by the time between the studies. Further covariates included in the analyses of both two studies were sociodemographic variables (i.e., age, gender, and education level [low, medium, and high]), health behaviour variables (i.e., current smoking status [yes/no] and use of alcohol [number of glasses per week]), and waist circumference (as a continuous variable). Education was slightly differently assessed in NESDA and DELTA but distinguished between low ((several years of) primary education), middle (secondary special education, lower secondary education, middle secondary education), and high (lower tertiary education, higher secondary education, middle tertiary education, bachelor’s degree at the university or higher or equivalent level) education.

In secondary analyses, as a check analyses were adjusted for use of antidepressants (yes/no), use of lipid‐modifying drugs (i.e., statins, yes/no), use of n‐3 PUFA supplements, and baseline depression severity score. As data on use of n‐3 PUFA supplements (yes/no) at baseline were only available in NESDA and one of the DELTA studies, only NESDA analyses were additionally adjusted for n‐3 PUFA supplement use. In all studies, medication and supplement use (NESDA only) were derived from drug container inspection and were considered as these could affect PUFA blood levels 45. Antidepressant medication (yes/no) involved tricyclic antidepressants (TCA), selective serotonin reuptake inhibitors (SSRI), and other antidepressants.

### Statistical analyses

Descriptive and frequency statistics were used to describe the study samples.

The datasets of the two DELTA studies were merged on the individual patient level. In the merged DELTA dataset, first undetectable fatty acid values (ranging between 2 (1.7%) and 44 (37%) non‐detectable levels per fatty acid) were imputed with the lowest detectable value divided by two, a method often used 46. Next, to impute missing values in covariates, one hundred imputed datasets were generated using Fully Conditional Specification (FCS, an iterative Markov chain Monte Carlo method) with IBM SPSS statistics software, version 22 (IBM Corp., Armonk, NY, USA). All confounders included in the analyses were imputed or used as predictors. Results from the analyses of the 100 separate datasets were pooled, correcting the standard errors of the regression coefficients for within‐imputation and between‐imputation variability.

In the two DELTA datasets, the resulting merged DELTA dataset and the NESDA dataset separately, proportional hazard approach to survival analysis (Cox regression analysis) was used with time until MDD recurrence (in months) as dependent variable and each of the FA measures at baseline as independent variables (in separate analyses). Hazard ratios (HRs), their corresponding 95% confidence intervals (95% CIs), and *P*‐values were reported. FA measures were z‐transformed to help comparing the estimates of the Cox regression models with different fatty acids as independent variables. Unadjusted Kaplan–Meier curves were presented to inspect the survival functions over time. All Cox regression models were adjusted for study wave (NESDA) or cohort study (DELTA), age, sex, education, smoking, alcohol, and waist circumference. Hereafter, it was tested whether antidepressant use, statin use, n‐3 PUFA supplement use (only in NESDA), and baseline depression severity scores had an individual effect on the association between fatty acids and recurrence of MDD.

The proportional hazard assumption was tested using Cox regression analyses with the log function of time until recurrence in months as dependent variable, and a categorical fatty acid measure and a categorical fatty acid measure‐by‐time interaction term as independent variables, for all fatty acid measures in separate analyses 47.

To check for consistency of findings, results from the Cox regression analyses in the NESDA and the merged DELTA study were pooled using random‐effects meta‐analyzing techniques. These analyses formed the main analyses of this paper. EPA was only measured in the DELTA studies, so secondary analyses examining associations between EPA and time until onset of MDD were thus only done in the DELTA studies. As some previous studies have shown that the association between FAs and depression (characteristics) might be different for males and females 30, in secondary analyses it was checked whether gender was an effect modifier, by including FA‐by‐gender interaction terms. To inspect non‐linear associations, as found before 48, 49, 50, 51, 52, quadratic associations were tested in secondary analyses. As additional secondary sensitivity analyses, fatty acid measures were divided into quartiles and associations with time until MDD recurrence were tested. Statistics (HR, 95% CI, and *P*‐values) per quartile were shown instead of overall trends because of our interest in possible non‐linear associations between FA measures and time until MDD recurrence. As additional check, analyses were repeated in a subsample of remitted participants that suffered from at least two previous major depressive disorder episodes (i.e., recurrent major depressive disorder (r‐MDD), *n* = 137), because this group has a higher recurrence rate than after one major depressive disorder episode 53.

All Cox regression analyses were conducted using IBM SPSS statistics software, version 22 (IBM Corp., Armonk, NY, USA). The significance level was set at 0.05. To correct for multiple testing, the Benjamini–Hochberg false discovery rate method was used based on 6 tests per model for NESDA and 7 for DELTA 54. The R packages ‘rmeta’ and ‘forestplot’ were used to pool results of the NESDA and DELTA and to visualize results (R Foundation for Statistical Computing, Vienna, Austria, 2016; https://www.R-project.org/).

## Results

### Descriptives of the study samples

Descriptive statistics of the studies can be found in Table 1. The mean age of the participants in the studies was rather comparable, and in all studies, the majority was female. Median number of previous MDD episodes during lifetime was 1 in NESDA and 4 in the merged DELTA study. In the total NESDA sample (*n* = 356), 98 NESDA participants (27.5%) had an MDD recurrence over a 6‐year period, while in the sample with recurrent MDD (r‐MDD) NESDA participants with at least two previous MDD episodes (*n* = 137), 43 participants (31.4%) had an MDD recurrence over a 6‐year period. In the merged DELTA sample, 67 participants (56.8%) had an MDD recurrence over an 8‐year period. Mean follow‐up durations were 63.2 months (SD = 21.4) in NESDA and 56.7 months (SD = 33.3) in the merged DELTA sample.

**Table 1:** Descriptive statistics of the study samples of NESDA and DELTA

	NESDA (*n* = 356)	DELTA‐1^st^ cohort (*n* = 75)	DELTA‐neuroimaging (*n* = 43)
Sociodemographics			
Age, mean (SD)	42.8 (12.6)	46.1 (9.05)	52.3 (7.84)
Female, *n* (%)	249 (69.9)	57 (76.0)	29 (67.4)
Education levels			
Low, *n* (%)	10 (2.8)	26 (34.7)	16 (37.2)
Middle, *n* (%)	166 (46.6)	26 (34.7)	19 (44.2)
High, *n* (%)	180 (50.6)	20 (26.7)	9 (18.6)
Lifestyle			
Smoking, yes, *n* (%)	146 (41.0)	15 (20.0)	8 (18.6)
Alcohol (# of glasses), median (p25‐p75)	3.74 (1.04–8.72)	2.00 (0.00–8.00)	4.88 (0.44–12.0)
Somatic health			
Waist circumference, median (p25‐p75)	86.0 (79.0–95.0)	87.0 (80.0–96.0)	94.5 (86.0–106.0)
Medication or supplement use			
Antidepressant use, yes *n* (%)	71 (19.9)	41 (54.7)	0 (0.0)
Statin use, yes *n* (%)	17 (4.8)	4 (5.3)	7 (16.3)
N‐3 PUFA supplement use, yes (%)	16 (4.5)	‐	6 (14.0)
MDD characteristics			
Residual symptoms on IDS‐SR_30_, median (p25‐p75)	9.00 (5.00–11.0)	‐	‐
Residual symptoms on HDRS, median (p25‐p75)	‐	3.00 (1.00–5.00)	2.00 (1.00–5.00)
Time since last MDD episode (months), median (p25‐p75)		26.0 (11.3–33.8)	24.0 (6.00–55.0)
Between 1 and 6 months ago, *n* (%)	49 (13.8)		
Between 6 months and 12 months ago, *n* (%)	26 (7.3)		
More than 12 months ago, *n* (%)	213 (59.8)		
Number of previous MDD episodes during lifetime			
Median (p25‐p75)	1.00 (1.00–3.00)	5.00 (3.00–10.0)	3.00 (2.00–5.00)
Number of participants with MDD relapse (the event)	98 (27.5)	41 (54.7)	26 (60.5)
Cumulative follow‐up time to event/censoring (in months)†	18 192	3145	691
Incidence rate (per 100 persons‐months)‡	0.54	1.30	3.76
Follow‐up duration in months, mean (SD)	63.2 (21.4)	67.6 (33.7)	29.6 (1.70)
Fatty acids			
DHA, median (p25‐p27)§	0.14 (0.11–0.17)	14.4 (11.9–17.5)	23.9 (17.1–29.1)
EPA, median (p25‐p75)§	N.A.	3.10 (2.20–4.30)	3.80 (2.80–5.50)
Total n‐3 PUFA, median (p25‐p75)§	0.38 (0.32–0.47)	27.8 (24.5–32.3)	41.8 (35.1–50.6)
Total n‐6 PUFA, median (p25‐p75)§	3.93 (3.43–4.44)	162.4 (157.4–178.8)	181.5 (173.8–189.7)
n‐3:n‐6 PUFA ratio, median (p25‐p75)§	0.10 (0.08–0.11)	0.15 (0.12–0.17)	0.22 (0.19–0.25)
Chain length index, median (p25‐p75)§	17.3 (17.1–17.6)	18.4 (18.4–18.5)	18.7 (18.6–18.8)
Unsaturation index, median (p25‐p75)§	1.23 (1.18–1.26)	1.33 (1.31–1.35)	1.44 (1.39–1.45)

SD, standard deviation; p25‐p27, 25^th^ percentile–75^th^ percentile; MDD, major depressive disorder; N.A., not applicable.

† Cumulative follow‐up time until event/censoring is the sum of all time until event/censoring for all participants, expressed in months.

‡ Incidence rate: number of participants developing disease/time to event/censoring in months * 100.

§ FA levels in NESDA are expressed in mmol/L and in the DELTA studies in pmol/10^6^ erythrocytes.

### Pooled findings of the association between FA measures and MDD recurrence

**Figure 1:**
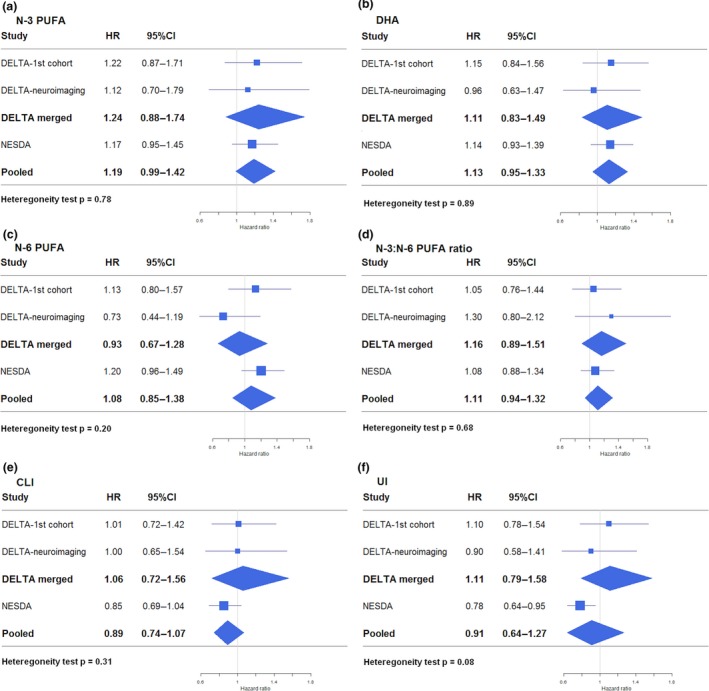
Forest plots showing fully adjusted associations of FA measures with MDD recurrence in the DELTA studies, the merged DELTA study, and the NESDA study, and the random‐effects pooled results. [Colour figure can be viewed at http://www.wileyonlinelibrary.com/]

**Figure 2:**
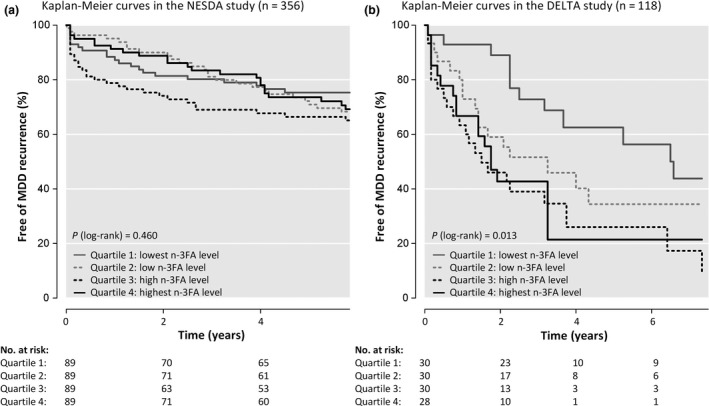
Kaplan–Meier curves in (a) the NESDA study (*n* = 356) and (b) the merged DELTA study (*n* = 118). NESDA (*N* = 356): *n* = 98 recurrences, *n* = 47 loss to follow‐up, and *n* = 211 administrative censoring. DELTA (*N* = 118): *n* = 67 recurrences, *n* = 24 loss to follow‐up, and *n* = 27 administrative censoring.

As shown in Fig. 1 and Table S3, none of the FA measures were significantly associated with time until MDD recurrence after pooling of the results. Additional adjustment for antidepressant use, statin use, n‐3 PUFA supplement use, and baseline depression severity did not change results (results available on request). All proportional hazard assumptions were met, except for n‐6 PUFA levels in NESDA, meaning that the ratio of the hazards for any two individuals was not constant over time and the one hazard provided in the table is not representative for the whole time period. Unadjusted Kaplan–Meier curves to inspect the survival functions over time are presented in Fig. 2.

### Secondary analyses

High EPA levels, which were only measured in the merged DELTA study, were significantly associated with a higher hazard on MDD recurrence (fully adjusted model: HR: 1.37, 95% confidence interval: 1.04–1.78, *P *= 0.023). In the merged DELTA study, a significant interaction term of gender with n‐6 PUFA level was found, while in NESDA and in the pooled results, no significant interactions with gender were found. After pooling the results, none of the squared FA measures to test for non‐linear effects were significant. Table S4 shows the association between FA quartiles and MDD recurrence, which confirmed there is no clear pattern of association between these FA measures and MDD recurrence. When Cox regression analyses were repeated in a smaller NESDA sample including only remitted MDD patients with ≥2 previous MDD episodes (*n* = 137), results did not change (results available on request).

## Discussion

Using a large and unique dataset combining two detailed, longitudinal cohorts, the present study investigated whether FA measures were prospectively related to MDD recurrence in remitted MDD patients. Results showed no protective effect of high n‐3 PUFA levels, high DHA levels, high EPA levels, low n‐6 PUFA levels, high n‐3:n‐6 PUFA ratios, long FA carbon chain length, or high degree of unsaturation for MDD recurrence in remitted MDD patient over a maximum of 8 years of follow‐up. These results remained when limiting analyses to a high‐risk group of remitted MDD patients with a least two previous MDD episodes. Overall, findings were consistent across both genders. To the best of our knowledge, to date no studies have examined the impact of FA measures on MDD recurrence specifically in remitted MDD patients or remitted recurrent MDD (r‐MDD) patients. These results suggest that alterations in FA levels noted in remitted MDD patients 24, 25 cannot be used as biomarkers of prognosis or targets of preventive interventions.

The results of this study are in line with the results of two previous studies on the association between n‐3 PUFA levels or intake and prospective MDD recurrence, which also found no evidence for such an association 30, 31. However, our results are not in line with one study that did find an association between n‐6:n‐3 PUFA blood ratios and prospective onset of MDD over 7 years 29. Also in our own previous longitudinal analysis by Thesing et al. (submitted) on partly the same population, we did not find that baseline PUFA levels were predictive of time until onset of a new depressive episode in participants with and without a history of MDD.

Because n‐3 PUFAs are associated with processes that are associated with MDD recurrence, such as dysregulation of the HPA axis 6, 9 and high scores on neuroticism 55, and some studies found lower n‐3 PUFA levels in remitted depressed patients compared to healthy controls 24, 25, it was hypothesized that these low n‐3 PUFA levels would be predictive for MDD recurrence. However, the results of our study could not confirm this. One explanation for the lack of association between PUFAs and MDD recurrence found in the present study might be that n‐3 PUFA alterations in remitted patients are an adaptive process, as described by Assies et al. (2014). As fatty acids are vulnerable for oxidation, lowering of fatty acids levels may be an adaptive mechanism 7. This may also explain (i) our finding that high EPA was associated with a shorter time until recurrence, and (ii) the finding that a large RCT testing the preventive effect on depression onset of a multi‐nutrient intervention (including n‐3 PUFAs) saw higher depressive and anxiety symptoms for supplements relative to placebo 32. If lower n‐3 PUFA levels during remission are an adaptive process, supplementation can be harmful instead of helpful.

This study has several limitations and strengths. First of all, although MDD recurrence was assessed repeatedly during follow‐up, recurrences were assessed retrospectively, and may therefore (mainly in NESDA and the first DELTA cohort study) be influenced by memory bias. However, the LCI (used in NESDA) uses a calendar method to determine life events during the 2‐year follow‐up period to refresh memory, which minimizes recall bias. More frequent follow‐up measures would have further decreased the risk for recall biases. Fatty acids in NESDA and DELTA were measured using two different assays; however, by running meta‐analyses with random effects, we acknowledged clustering of measures within cohorts. As there is no universally agreed/golden standard reference range for PUFA levels, and as this also depends on measurement aspects including laboratory‐specific analytical procedures, a reference range for low vs. high PUFA levels cannot be given or used in the current study. The results of this paper are not entirely independent of the results of our previous study by Thesing et al. (submitted), in which we focused on a smaller number of PUFA outcomes and found that baseline PUFA levels were not predictive of time until onset of a new depressive episode in a mixed sample of participants with and without a history of MDD. Life stress is a possible predictor of recurrence in recurrent depression, so inclusion of life stress as a predictor could have reduced the residual variance and may have increased the chance to find a predictive effect of PUFA measures. Aerobic exercise is an effective treatment for depression, and it may have an impact on PUFA concentrations too. It may have been interesting to investigate the relationships between exercise, PUFAs, and recurrence in remitted patients with recurrent depression. However, both life stress and aerobic exercise were not measured and fall beyond the scope of our current study. Future studies could investigate this. Finally, unfortunately, data on use of n‐3 PUFA supplements were lacking in one of the DELTA studies.

A strength of the present study is the unique and large combined dataset of two detailed, longitudinal cohorts and the use of an original sample with only remitted (and partly recurrent) MDD patients that has not yet been studied before. Another strength of the current study compared to previous studies is that next to absolute FA levels, also total FA metabolism measures were included, namely CLI and UI. In this study, these indices seemed to be the most informative (e.g., an association in the expected direction, although not significant). As CLI and UI are not only influenced by n‐3 and n‐6 PUFAs but also by saturated fatty acids and monounsaturated fatty acids, the CLI and UI may reflect overall fatty acid metabolism best.

Regarding clinical practice, our observational data do not support the use of n‐3 PUFA supplementation as a viable and recommendable option to prevent MDD recurrence in remitted MDD patients. Although n‐3 PUFA supplements have been shown to effectively influence several disorder‐related mechanisms, for example, inflammation 56, our data are in line with interventional evidence that shows that this does not result in a protective effect on MDD onset 32. However, it should be noted that preventive effects can be different from treatment effects during a current depressive episode, given that some meta‐analyses showed that n‐3 PUFA supplementation during an MDD episode may have antidepressant effects 18, although heterogeneity exists (Appleton et al., 2015; Carney et al., 2019; Sarris et al., 2016). Moreover, effect sizes for intervention studies can expected to be largest when focusing on the acute depressed state with a careful selection of patients, for example, according to inflammatory status 57. As potential biomarkers can apply to depression risk, diagnosis, state or acuity, stage, treatment response, or prognosis 19, 58, our suggestion for future studies is to look further on what kind of a biomarker FAs might be for depression. In addition, more detailed metabolic analyses may result in stronger prospective associations.

To summarize, this study is the first to examine the association of both baseline n‐3 and n‐6 PUFA levels and baseline PUFA indices with a detailed time until MDD recurrence measure over a maximum of 8 years of follow‐up, in a relatively large sample of remitted MDD patients, with the ability to additionally check consistency of findings in a high‐risk group of remitted r‐MDD patients with a least two previous MDD episodes. We found no protective/preventive effect of high n‐3 PUFA levels, high EPA levels, high DHA levels, low n‐6 PUFA levels, high n‐3:n‐6 PUFA ratios, long FA carbon chain length, and a high degree of unsaturation for MDD recurrence in remitted MDD patients, neither when restricting our analyses to only remitted r‐MDD patients. By combining two representative studies, we provide more definitive evidence that refutes the idea that FA alterations during remission of MDD pose a vulnerability marker for recurrence, which is of clinical relevance.

## Declaration of interest

AL, YM, JA, CB, CF, EG, HR, AS, MB and RM have no conflicts of interest to report.BP has received (non‐related) research funding from Jansen Research and Boehringer Ingelheim.

## Supporting information


**Table S1**. Spearman correlations between all fatty acid measures in NESDA (*n* = 356).
**Table S2**. Spearman correlations between all fatty acid measures in the merged DELTA study (*n* = 118).
**Table S3**. Associations between fatty acid measures and (time until) relapse of MDD using Cox regression analyses in the NESDA study (*n* = 356) and the merged DELTA study (*n* = 118) and the pooled results.
**Table S4**. Associations between fatty acid measures (divided into quartiles) and (time until) relapse of MDD using Cox regression analyses in the NESDA study (*n* = 356) and the merged DELTA study (*n* = 118).

## Data Availability

Upon request, a data transfer agreement can be drafted.
