# Supplementary material for: Fatty acids and recurrence of major depressive disorder: combined analysis of two Dutch clinical cohorts
Source: Acta Psychiatr Scand. 2019 Dec 26;141(4):362–73. doi: 10.1111/acps.13136 (PMC7216896; doi:10.1111/acps.13136)
Supplement: Supplementary file 1 — Table S1. Spearman correlations between all fatty acid measures in NESDA (n = 356). Table S2. Spearman correlations between all fatty acid measures in the merged DELTA study (n = 118). Table S3. Associations between fatty acid measures and (time until) relapse of MDD using Cox regression analyses in the NESDA study (n = 356) and the merged DELTA study (n = 118) and the pooled results. Table S4. Associations between fatty acid measures (divided into quartiles) and (time until) relapse of MDD using Cox regression analyses in the NESDA study (n = 356) and the merged DELTA study (n = 118). [file ACPS-141-362-s001.docx]

**Supplement**

Table S1. Spearman correlations between all fatty acid measures in NESDA (n=356)

|  | N-3 PUFA | DHA | N-6 PUFA | N-3:N-6 PUFA | CLI | UI |
| --- | --- | --- | --- | --- | --- | --- |
| N-3 PUFA | 1 |  |  |  |  |  |
| DHA | .87 ** | 1 |  |  |  |  |
| N-6 PUFA | .61 ** | .49 ** | 1 |  |  |  |
| N-3:N-6 PUFA | .78 ** | .73 ** | .03 | 1 |  |  |
| CLI | .02 | .17 ** | -.21 ** | .21 ** | 1 |  |
| UI | .03 | .05 | -.22 ** | .21 ** | .04 | 1 |
| *Note. *** <.01 | | | | | | |

Table S2. Spearman correlations between all fatty acid measures in the merged DELTA study (n=118)

|  | n-3 PUFA | DHA | EPA | N-6 PUFA | N-3:N-6 PUFA | CLI | UI |
| --- | --- | --- | --- | --- | --- | --- | --- |
| N-3 PUFA | 1 |  |  |  |  |  |  |
| DHA | .95 ** | 1 |  |  |  |  |  |
| EPA | .70 ** | .60 ** | 1 |  |  |  |  |
| N-6 PUFA | .20 * | .13 | -.09 | 1 |  |  |  |
| N-3:N-6 PUFA | .94 ** | .92 ** | .73 ** | -.12 | 1 |  |  |
| CLI | .76 ** | .69 ** | .28 ** | .29 ** | .67 ** | 1 |  |
| UI | .82 ** | .35 ** | .35 ** | .26 ** | .74 ** | .93 ** | 1 |
| *Note.* * <.05 ** <.01 | | | | | | | |

Table S3. Associations between fatty acid measures and (time until) relapse of MDD using Cox regression analyses in the NESDA study (n=356) and the merged DELTA study (n=118) and the pooled results.

|  | NESDA (n=356) | | | |  | Merged DELTA study (n=118) | | | |  | Pooled results | | | |
| --- | --- | --- | --- | --- | --- | --- | --- | --- | --- | --- | --- | --- | --- | --- |
|  | Time until first relapse of MDD | | | |  | Time until first relapse of MDD | | | |  | Time until first relapse of MDD | | | |
|  | HR | 95% CI lower level | 95% CI  upper level | p-value |  | HR | 95% CI lower level | 95% CI upper level | p-value |  | HR | 95% CI lower level | 95% CI upper level | p-value |
| N-3 PUFA  Unadjusted  Fully adjusted | 1.04  1.17 | 0.86  0.95 | 1.27  1.45 | .69  .15 |  | 1.41  1.24 | 1.13  0.88 | 1.78  1.74 | .003  .23 |  | 1.19  1.19 | 0.86  0.99 | 1.66  1.42 | .29  .063 |
| DHA  Unadjusted  Fully adjusted | 1.07  1.14 | 0.88  0.93 | 1.30  1.39 | .50  .21 |  | 1.33  1.11 | 1.05  0.83 | 1.69  1.49 | .017  .49 |  | 1.18  1.13 | 0.95  0.95 | 1.47  1.33 | .128  .158 |
| EPA  Unadjusted  Fully adjusted | NA  NA | NA  NA | NA  NA | NA  NA |  | 1.39  1.37 | 1.11  1.04 | 1.74  1.78 | **.004 ***  **.023 *** |  | NA  NA | NA  NA | NA  NA | NA  NA |
| N-6 PUFA  Unadjusted  Fully adjusted | 1.06  1.20 | 0.87  0.96 | 1.29  1.49 | .55  .105 |  | 1.11  0.93 | 0.85  0.67 | 1.45  1.28 | .45  .65 |  | 1.08  1.08 | 0.92  0.85 | 1.26  1.38 | .35  .52 |
| N-3:N-6 PUFA ratio  Unadjusted  Fully adjusted | 1.02  1.08 | 0.84  0.88 | 1.25  1.34 | .83  .46 |  | 1.30  1.16 | 1.05  0.89 | 1.61  1.51 | .015  .26 |  | 1.15  1.11 | 0.91  0.94 | 1.46  1.32 | .24  .20 |
| CLI  Unadjusted  Fully adjusted | 0.91  0.85 | 0.75  0.69 | 1.11  1.04 | .36  .116 |  | 1.33  1.06 | 1.06  0.72 | 1.68  1.56 | .014  .76 |  | 1.01  0.89 | 0.76  0.74 | 1.59  1.07 | .63  .21 |
| UI  Unadjusted  Fully adjusted | 0.81  0.78 | 0.67  0.64 | 0.97  0.95 | **.024 ***  **.014 *** |  | 1.33  1.11 | 1.06  0.79 | 1.68  1.58 | **.015 ***  .55 |  | 1.03  0.91 | 0.63  0.64 | 1.68  1.27 | .90  .57 |
| *Note*. Fatty acid measures are standardized. Fully adjusted models are adjusted for study wave (for NESDA: metabolic shipment wave 1 and 2) and cohort study study (for DELTA: DELTA 1^st^ cohort study vs. DELTA-neuroimaging), age, sex, education level (low, medium, high), current smoking status (yes/no), alcohol (glasses per week), and waist circumference. MDD: Major Depressive Disorder. HR: Hazard ratio. CI: Confidence interval. N-3: omega-3. PUFA: Polyunsaturated fatty acids. DHA: docosahexaenoic acid. EPA: eicosapentaenoic acid. N-6: omega-6. * Significant after correction for multiple testing according to the False Discovery Rate using the Benjamini-Hochberg method based on 7 tests (for NESDA and the pooled results) or 8 tests (for the merged DELTA study) per model. | | | | | | | | | | | | | | |

Table S4. Associations between fatty acid measures (divided into quartiles) and (time until) relapse of MDD using Cox regression analyses in the NESDA study (n=356) and the merged DELTA study (n=118).

|  | NESDA (n=356) | | | |  | Merged DELTA study (n=118) | | | |  | Pooled findings | | | | |
| --- | --- | --- | --- | --- | --- | --- | --- | --- | --- | --- | --- | --- | --- | --- | --- |
|  | Time until first relapse of MDD | | | |  | Time until first relapse of MDD | | | |  | Time until first relapse of MDD | | | | |
|  | HR | CI lower level | CI upper level | p-value |  | HR | CI lower level | CI upper level | p-value |  | HR | | CI lower level | CI upper level | p-value |
| N-3 PUFA |  |  |  |  |  |  |  |  |  |  |  | |  |  |  |
| Quartile 1 (ref.) |  |  |  |  |  |  |  |  |  |  |  | |  |  |  |
| Quartile 2 | 1.39 | 0.77 | 2.51 | .28 |  | 1.71 | 0.78 | 3.74 | .18 |  | 1.50 | | 0.93 | 2.40 | .094 |
| Quartile 3 | 2.05 | 1.13 | 3.72 | .018 * |  | 2.51 | 1.14 | 5.53 | .023 * |  | 2.21 | | 1.37 | 3.55 | .001 |
| Quartile 4 | 1.59 | 0.82 | 3.06 | .169 |  | 1.51 | 0.57 | 4.01 | .41 |  | 1.56 | | 0.91 | 2.69 | .109 |
| DHA |  |  |  |  |  |  |  |  |  |  |  | |  |  |  |
| Quartile 1 (ref.) |  |  |  |  |  |  |  |  |  |  |  | |  |  |  |
| Quartile 2 | 1.26 | 0.67 | 2.37 | .48 |  | 1.78 | 0.83 | 3.79 | .136 |  | 1.45 | | 0.89 | 2.35 | .13 |
| Quartile 3 | 2.03 | 1.14 | 3.64 | .017 * |  | 1.51 | 0.70 | 3.25 | .29 |  | 1.81 | | 1.15 | 2.90 | .011 * |
| Quartile 4 | 1.63 | 0.89 | 2.97 | .113 |  | 1.18 | 0.52 | 2.67 | .70 |  | 1.38 | | 0.83 | 2.30 | .21 |
| EPA |  |  |  |  |  |  |  |  |  |  |  | |  |  |  |
| Quartile 1 (ref.) |  |  |  |  |  |  |  |  |  |  |  | |  |  |  |
| Quartile 2 | NA | NA | NA | NA |  | 2.05 | 0.87 | 4.82 | .101 |  | NA | | NA | NA | NA |
| Quartile 3 | NA | NA | NA | NA |  | 1.59 | 0.62 | 4.08 | .33 |  | NA | | NA | NA | NA |
| Quartile 4 | NA | NA | NA | NA |  | 1.85 | 0.66 | 5.18 | .24 |  | NA | | NA | NA | NA |
| N-6 PUFA |  |  |  |  |  |  |  |  |  |  |  | |  |  |  |
| Quartile 1 (ref.) |  |  |  |  |  |  |  |  |  |  |  | |  |  |  |
| Quartile 2 | 1.35 | 0.75 | 2.41 | .32 |  | 1.27 | 0.64 | 2.54 | .50 |  | 1.31 | | 0.84 | 2.05 | .23 |
| Quartile 3 | 1.31 | 0.71 | 2.43 | .39 |  | 0.68 | 0.31 | 1.52 | .35 |  | 1.00 | | 0.53 | 1.87 | 1.00 |
| Quartile 4 | 1.71 | 0.92 | 3.18 | .093 |  | 0.54 | 0.22 | 1.32 | .18 |  | 1.00 | | 0.32 | 3.11 | 1.00 |
| N-3:N-6 PUFA |  |  |  |  |  |  |  |  |  |  |  | |  |  |  |
| Quartile 1 (ref.) |  |  |  |  |  |  |  |  |  |  |  |  | |  |  |
| Quartile 2 | 1.10 | 0.62 | 1.97 | .75 |  | 1.08 | 0.47 | 2.47 | .85 |  | 1.09 | 0.68 | | 1.76 | .71 |
| Quartile 3 | 1.55 | 0.88 | 2.75 | .13 |  | 2.24 | 0.95 | 5.26 | .065 |  | 1.74 | 1.08 | | 2.80 | .022 * |
| Quartile 4 | 1.21 | 0.65 | 2.23 | .55 |  | 1.01 | 0.41 | 2.46 | .99 |  | 1.14 | 0.69 | | 1.89 | .62 |
| Chain length index |  |  |  |  |  |  |  |  |  |  |  |  | |  |  |
| Quartile 1 (ref.) |  |  |  |  |  |  |  |  |  |  |  |  | |  |  |
| Quartile 2 | 0.77 | 0.45 | 1.35 | .36 |  | 0.83 | 0.43 | 1.63 | .60 |  | 0.78 | 0.52 | | 1.22 | .29 |
| Quartile 3 | 0.50 | 0.27 | 0.91 | .023 * |  | 1.97 | 0.67 | 5.80 | .22 |  | 0.92 | 0.24 | | 3.55 | .91 |
| Quartile 4 | 0.70 | 0.40 | 1.22 | .205 |  | 1.32 | 0.29 | 6.08 | .72 |  | 0.75 | 0.44 | | 1.27 | .29 |
| Unsaturation index |  |  |  |  |  |  |  |  |  |  |  |  | |  |  |
| Quartile 1 (ref.) |  |  |  |  |  |  |  |  |  |  |  |  | |  |  |
| Quartile 2 | 0.58 | 0.34 | 1.01 | .055 |  | 1.42 | 0.74 | 2.74 | .30 |  | 0.89 | 0.37 | | 2.14 | .80 |
| Quartile 3 | 0.63 | 0.36 | 1.08 | .090 |  | 0.98 | 0.40 | 2.37 | .96 |  | 0.71 | 0.44 | | 1.12 | .14 |
| Quartile 4 | 0.40 | 0.22 | 0.74 | .004 * |  | 0.78 | 0.26 | 2.33 | .66 |  | 0.48 | 0.27 | | 0.86 | .014 * |
| *Note.* Models are adjusted for study wave (for NESDA: metabolic shipment wave 1 and 2) and study (for DELTA: DELTA first cohort study vs. DELTA-neuroimaging), age, sex, education level (low, medium, high), current smoking status (yes/no), alcohol (glasses per week), and waist circumference. MDD: Major Depressive Disorder. HR: Hazard ratio. CI: Confidence interval. N-3: omega-3. PUFA: Polyunsaturated fatty acids. DHA: docosahexaenoic acid. EPA: eicosapentaenoic acid. N-6: omega-6. * Significant after correction for multiple testing according to the False Discovery Rate using the Benjamini-Hochberg method using 18 (NESDA and pooled results) or 21 (DELTA results) tests. | | | | | | | | | | | | | | | |
